# Mr. Ring's Cases of Secondary Small-Pox

**Published:** 1807-02

**Authors:** John Ring


### *To the Editors of the Medical and Physical Journal.*

Gentlemen,

I Beg leave to communicate some cases of the small-pox occurring a second time, in addition to others which I have before transmitted.

### *Cases of secondary Small-pox.*

Mrs. Turton, of Long Parish, near Andover, has twice had the small-pox; and was each time blind with the disorder. This case was communicated to me by her nephew, Mr. Turton, No. 57, Swallow Street.

Mrs. Jones, No. 2, James Street, Manchester Square, was inoculated for the small-pox, when six months old, by by the late Mr. Stredwick of Richmond; who assured her parents, that the operation had succeeded, and that she was perfectly safe. Her arms rose; and proper scars remain. When twelve years old, she went to see a man labouring under the disease; who died of it the next day. She thought herself in no danger on this account; having been with three of her brothers and sisters when under inoculation; and having escaped infection. She now, however, caught the disorder; had it severely, and was blind two days.

Miss Weller, No. 6, Panton Street, was inoculated for the small-pox at Tunbridge, in her infancy, by Mr. Fossart; when her brother had it in the natural way very full, and was blind. She was with him the whole time of the disorder. In the beginning of last June, she had the disease again in the natural way; and was affected with the usual symptoms for the space of three days, before the eruptions began to appear. They first shewed themselves on the face; and gradually descended to the lower extremities. She has two very distinct scars on her arm, in consequence of inoculation. In this last illness, she was a patient of Mr. Anderson, of Fleet Street; who did me the favour to inform me of the case. It was also seen by Dr. Jenner, Mr. Paytherus, Mr. John Ring, and my assistant, Mr. Batho.

The idea, that a person can have the small-pox but once, has certainly been very prevalent, and even general in this country; but it appears to be erroneous, and is opposed by authorities full as respectable as those by which it has been supported. In the Monthly Review for March, 1806, are the following observations. "The remarks of Burserius may be considered as the more valuable, because he cannot be suspected of any undue bias. *He quotes instances of the small-pox occurring more than once,* from some of the older writers, and then adds, But, for the sake of brevity, passing over the numerous testimonies of foreigners, I shall only touch upon a few of those of the Italian physicians, that I may not seem to lose sight of such as are afforded by our own writers. It is not an uncommon thing in Naples, as we are informed by Sarcon and Mosca, for the same person to be attacked twice or thrice with the small-pox; and of the confluent kind.

"The same observation holds good in Florence: Targioni, in the year 1775, saw a woman twice attacked with the small-pox in the natural way; and in the following year he published another account of the recurrence of the small-pox.

small-pox. Juvanelli attended three sisters in the small-pox at the same time; who, on a former occasion, had laboured under the general complaint; as it was acknowledged to be by the physicians who attended them.

"In like manner Lilius, in order to establish the fact, *published two complete histories of the recurrence of the small-pox*, in the year 1777. I find nearly the same opinion entertained by the ingenious Azzoquidi, Professor of Medicine at Bologna; who, not content with mentioning two instances of the recurrence of the complaint, adduces the case of an old woman, who, as we are informed by Borelli, at the age of 118, died of the eighth attack of the small-pox.

"Lastly, to crown the whole, he mentions the case of Louis XV; who, after experiencing the complaint at the age of 14, was afterwards attacked with it at the age of 64. I might confirm the fact by the observations published by Girard, then residing at Padua, as well as by other testimonies, were I not restrained by a great controversy, which shortly after arose concerning them."

---

The Royal College of Physicians of Edinbnrgh having elected Dr. Jenner an Honorary Fellow, I have obtained permission of Dr. Spens, the President of the College, to publish his letter announcing that election, together with the extract from the minutes of the College which accompanied it, in any way which I might judge most conducive to the important end in view.

*Copy of a Letter from Dr.* Spens *to Dr.* Jenner, *dated Edinburgh, May* 20, 1806.

"Sir,

"In the absence of Dr. James Home, Secretary of the Royal College of Physicians of this city, I have the honour to inform you, that at a meeting held this day, and for the express purpose, they unanimously elected you an Honorary Fellow of their College.

"This mark of distinction, with very few exceptions, has for the last fifty years been confined to foreign physicians of the first eminence.

"The College have conferred it on you, in testimony of their conviction, of the immense benefits which have been, and will, they trust, in all future ages, be derived to the world, from inoculation for the cow-pox; and as a mark of their sense of your very great merits and ability, in introducing, and promoting, this invaluable practice.

"As

"As an individual, allow me to express the satisfaction I feel, in conveying to you the sentiments of the body to which I belong; and in which, at present, I have the honour to preside.

"I have the honour to be, Sir,
Your most obedient humble servant,
THOMAS SPENS, C. R. M. ED. PR."

"*Dr. Edward Jenner.*"

*To this Letter of Dr.* SPENS, *I beg leave to subjoin the following extract of one from Dr.* STUART, *a Fellow of the College, to Dr.* JENNER, *dated August* 19, 1806, *relative to the same subject.*

"The Diploma of the College will soon be sent to you; and it will give every one of us satisfaction, that it be regarded as expressing our full assurance of the safety, and immense utility, of Vaccination.

"The state of my health has, in a great measure, removed me from practice; but from the testimonies of other practitioners, and the proofs of its salutary consequences, at the Public Dispensary, where I still attend, I am satisfied, that if all the world could be persuaded to inoculate with cow-pox matter, more blessed results would arise to mankind, than have hitherto been produced by medicine and surgery in general.

"About 1200 have been inoculated at the Dispensary since January last; and 6468 previous to that time, since the commencement of the practice there; and you may say confidently, that with the utmost care, in attending to every report unfavourable to the practice, in no one instance has any thing appeared, which does not recommend it for universal adoption.

"I was surprised at seeing it mentioned in the newspapers, as meeting with opposition in Scotland. The reverse is the case, I believe, almost universally. I shall be most happy, if Government should express the national gratitude, in a different way from what they have hitherto done; and such, I trust, is the sentiment of every one among us, who has observed, and considered the practice. It would give me pleasure to answer any inquiries, on the subject of vaccination; or to do any thing in my power, to promote the practice."

---

These testimonials in favour of Dr. Jenner, and his valuable discovery, do equal honour to him, and to that celebrated brated source of medical science, from which they flow. It would, however, be unreasonable to expect, that every thing which flows from that source should be equally pure; or that every document which comes from Edinburgh, should be equally respectable. One gentleman belonging to that seat of learning, who is the editor of a Journal, talks of the acrimony of others, and takes care to give some pretty good specimens of his own. He talks of partisans; and acts the part of a partisan. He censures others, for interlarding their writings with something like wit. This is an offence, which he himself never has committed; and never will.

This gentleman is said to furnish materials for the medical articles of the Edinburgh Review; and to have given an instance of *his temperate and impartial manner* of investigating controverted subjects, in his review of Thompson's Chemistry. The materials, thus furnished, are, indeed, wrought into form by an abler hand.—Whether he furnished the materials for the late article on vaccination, I know not; but, whoever furnished them, I beg leave to correct some erroneous statements which they contain.

It is there asserted, that previously to Dr. Jenner's communicating his discoveries to the world, he had vaccinated some hundreds of children; and put them to the test of variolous inoculation. This is a mistake; when Dr. Jenner published an account of his discovery, he had not vaccinated many persons; nor would it have redounded to his credit, to have delayed his publication longer; since, by imparting it to the world, he enabled others to assist him in his inquiries, and to accelerate the propagation of this beneficial practice.

The next error is a trivial one. Mr. Cooper is called Ashley instead of Astley.

It is then remarked, that "some *candid* and interesting discussion, as to the symptoms and effects of the disease, took place between Dr. Jenner, and Drs. Woodville and Pearson."—It is, however, very well known to every one, who is in the least degree acquainted with the history of vaccination, that the discussion here alluded to was not always *candid*.—The honey-moon of vaccination was soon over; and the few cold compliments which Dr. Jenner at first received from his rivals in the metropolis, were succeeded by the language of invective and reproach.

It is then asserted by the Reviewer, that under the superintendance of Drs. Woodville and Pearson, the practice of vaccination was prosecuted to a great extent.—He forgets, forgets, however, to mention, that under their superintendance, the small-pox was propagated instead of the cow-pox; and that the reports which they published, and the matter which they disseminated, were such as created a very general prejudice against the practice. He also forgets to mention, that this contaminated matter was in general sold at a very high price.

It was on this account, the author of these remarks embraced an early opportunity of procuring matter from the stock of Dr. Jenner; which he has from that time diffused in every direction; and sent it to many thousands of practitioners, in various parts of the world. In particular he furnished with it, some of the principal inoculators in the metropolis; who, till that period, were in the habit of using and distributing matter which produced pustular eruptions. Some account of these eruptions, and of the confusion occasioned by them, may be seen in my Treatise.

He also devoted a considerable portion of his time to the gratuitous vaccination of the poor, partly at his own house, and partly at their own; a plan which, for obvious reasons, is better calculated to succeed than any other; but which a professional man cannot always pursue, on account of his other duties and avocations.

The Reviewer represents Messrs. Blair and Merriman, and the author of these remarks, as answering every body; and accuses them of intemperance and personality. How far these accusations are just, the world will judge. To me it certainly appears, that this complaint commonly originates with those who smart under the lash of criticism; and is echoed by others, who have never taken the trouble to examine how far those criticisms are well founded.

Whatever may be the demerits of the author of these remarks, he cannot subscribe to the censure passed by the Reviewer on his fellow-labourers; and in this opinion he is far from being singular. The two publications in question, have received ample applause from some of our best English critics; and it was reserved for the keen eyes of the Edinburgh Reviewer to find in them such palpable defects. Mr. Merriman's publication has, for the most part, been considered as temperate; and if Mr. Blair's appeared otherwise to the Reviewer, it was probably from his not reading enough of it to discover, that the severe passages are, in general, not originally his own; but answers to Dr. Rowley, borrowed from his own publication. But such a neglect is no wonder, while we are insulted by public public advertisements for scribblers of this cast, who are to do their work without cutting open a book!

As the doctrine of a temporary prophylactic still has some advocates, I shall transcribe the opinion of the Reviewer on that head; leaving it to every one to attach as much, or as little importance to that opinion, as he thinks proper. My own opinion is, that with respect to the authors who have written on the subject of vaccination, he is in some measure prejudiced by the misrepresentations of a *partisan*; but, that he has treated the question itself with considerable ability. His remarks on the subject of a temporary prophylactic are as follows.

"Mr. Goldson's theory need not detain us long. It exhibits, we think, as perverse an application of scepticism and credulity, as we have ever met with. There are perhaps one hundred authenticated cases of the natural cow-pox, in which the patients have been found to resist variolous infection; and upon this scanty testimony, Mr. Goldson implicitly believes, that the natural cow-pox is an infallible preventive of the small-pox. There are more than one hundred thousand cases of the inoculated cow-pox, in which the patients have equally resisted all subsequent infection; and yet he refuses to believe, that the inoculated cow-pox can be depended on as a preventive! This is almost as absurd, as it is in Mr. Birch, first to tell us, that the cow-pox is nothing but the small-pox transmitted through a cow; and then to maintain, that it is in the highest degree hazardous and improper, to substitute the cow-pox inoculation for that of the small-pox. *Yet these are the two most rational antagonists of vaccination.*

"Dr. Moseley's notion, however, we believe, has had more currency; and certain timid persons have been induced to suspect, that the security afforded by vaccination is not of a permanent nature; but liable to be exhausted by time. *It is certain, however, that there is nothing either in Dr. Moseley's reasonings, or in the analogy of other physiological facts, to justify such an observation.* Dr. Moseley says, there are many eruptive diseases, which render the constitution, for the time, incapable of variolous infection, though they were never understood to impart any permanent security.—*Now, we admit the premises upon which this reasoning is founded; but we utterly deny the conclusion. We believe it to be perfectly well established, that certain violent cases of eruption will often indispose the body to receive the infection of the small-pox; and enable it indeed, for the time, to resist every species of cutaneous infection;*

*infection; but it is necessary to observe, that this effect is only produced, during the actual presence and continuance of the eruptive distemper.—It was never pretended by any body, not even by Dr. Moseley, that a person completely cured of the scald head, or the itch, or the leprosy, would resist the infection of the small-pox, merely because he had been affected with these distempers, some months or years before. If the cow-pox, therefore, have no other preventive virtue than these disorders, then it ought only to resist the small-pox, during the fifteen or twenty days that the vesicle continues; and the patient must again be liable to contagion, in less than a month.*"

While a single adherent to Mr. Goldson's opinion remains, no friend of vaccination will think a few moments mis-spent in discussing the subject, and removing any vestige of doubt, that may still remain, from the minds of the public.—It may be said, that failures, or at least apparent failures, have occurred, in the inoculated cow-pox; but it is certain, that failures, or at least apparent failures, have also occurred in the casual disease.

The Reviewer has displayed equal ability in his comparative view of the security arising from the inoculation of the small-pox. He says, the occurrence of the small-pox, after variolous inoculation, is a circumstance which *the patrons of that practice have been much disposed to keep out of sight.* "The general rule," he observes, certainly is, that no person has the disease twice; and in a certain sense, the exceptions to it must be allowed to be very few indeed; but it is an established fact, that very many persons who have gone through the disease, either in the natural way, or by inoculation, are liable, when inoculated a second time, or exposed to powerful contagion, to a second and mitigated attack of fever and eruptions; in the course of which pustules are formed; from which the genuine small-pox may be inoculated.

"Nurses, who sleep with children in the small-pox, are familiarly known to be liable to these affections; and that, many times in the course of their lives. A multitude of indisputable cases are cited in the volumes before us, of similar effects produced by a second inoculation for the small-pox, after the first has taken full effect;* or even after

" See Ring's Answer to Moseley, and authorities cited, p. 194, &c.—Moore's Reply, p. 55, 56, &c.—Willan, p. 65, 71, &c.—Birch's Serious Reasons, p. 45."

after a very severe and dangerous attack of the natural disorder. In all these cases, however, the symptoms are decidedly milder than in the proper original small-pox. The fever is of shorter duration; and the pustules are smaller; and dry up, and fall off much earlier, than in the genuine form of the disease. There is no instance, in which it has been followed by fatal or serious consequences. Now, it is apparent, even upon the face of the statements made by the antivaccinists themselves, that almost all the alleged cases of small-pox following vaccination, were cases of this description. The fever was always shorter than usual; the pustules were smaller, and usually fewer in number; and almost in every case, without exception, they were found to dry up and disappear much sooner than in the true original disorder."

After observing, that it seems to be confirmed by the import of the whole evidence, that vaccine inoculation bestows at least as great security as the inoculation of the small-pox, and that, even when adopted after infection is received, it appears to modify and controul the small-pox, so as to deprive it of all its alarming malignity, the Reviewers proceed as follows:

"With these cautions and observations, we may safely leave the reader to peruse all the cases which are detailed by the enemies of vaccination, in evidence of its inefficiency as a preventive of small-pox. Of the temper and judgment with which they are selected and narrated, some conjecture may be formed from the specimens which have been already given of their writing; but there is one sentence of Dr. Rowley's which seems to render any further observation unnecessary, and to make it superfluous to hunt through the laborious and persevering detection of the vaccinists. This learned Doctor, who has collected many more cases of failure than all his brethren put together, disposes of the whole controversy in this peremptory manner.

"Indeed, no other questions are admissible in vaccination, than, Have the parties been *inoculated* for the cow-pox? Have they been vaccinated? *Yes.* Have they had the small-pox afterwards? *Yes.* As to *how, when, where, whether the cow-pox took, was genuine, or spurious, or any arguments, however specious,* as pretexts for doubt or failure, they are evasive and irrelative to the question. They may confound fools, but not heighten the credit of vaccination." p. 34.

"After such a declaration, it certainly cannot be worth while to refute Dr. Rowley's cases. It would be little less absurd to tell a Jury, in a trial for murder, that the only question was, whether a pistol had been fired or not, and that it was of no consequence to inquire, whether it was loaded with ball, or whether the sufferer had died by a pistol shot.

"The antivaccinists themselves seem to admit, that by such *irrelative* and *evasive* inquiries, more than nine tenths of their cases of failure may be explained in a satisfactory manner. But still, they urge, there are a few remaining, which have been admitted by the vaccinators themselves to have exhibited the decisive symptoms of genuine cow-pox, followed by genuine small-pox. The admission to which these gentlemen allude, is contained in the first of the following paragraphs of a Report given in by the Medical Council of the Jennerian Society, and signed by upwards of fifty of the most eminent practitioners in London. That Council, upon considering the Report of a Committee, declare, that it appears to them, among other things,

'That it is admitted by the Committee, that a few cases have been brought before them, of persons having the small-pox, who had *apparently* passed through the cow-pox in a regular way.

'That cases, supported by evidence equally strong, have been also brought before them, of persons who, after having once regularly passed through the small-pox, either by inoculation or natural infection, have had that disease a second time.

'That in many cases, in which the small-pox has occurred a second time, after inoculation or the natural disease, such recurrence has been particularly severe, and often fatal; whereas, when it has appeared to occur after vaccination, the disease has generally been so mild, as to lose some of its characteristic marks, and even sometimes to render its existence doubtful.'

"Now, there are two ways of viewing this, equally reconcileable with the facts of the case, and with the report of the society. The one is, to hold that though those few persons *appeared* to have gone through the cow-pox regularly, yet that, in reality, there had been something imperfect in the vaccination; and that, if the means of more exact scrutiny had been afforded, such an imperfection might have been made manifest. This is the decided opinion of Mr. Moore, who says, 'he can more easily believe that an able able physician should commit a mistake (or disguise one) than that such an incongruity should occur;' and of Dr. Willan, who, after stating the result of his own most careful observation to be uniformly in favour of the claims of vaccination, says, 'If such failures do ever occur, they must occur in a very small proportion; and I am convinced that the subjects of them will not be found liable to take small-pox in the same manner and form as before vaccination.' The other view of the question is, that these failures do really occur, but in so very small a proportion, as to furnish no objection whatsoever against the practice of vaccination. That practice must maintain its ground triumphantly, if it can be shewn to be *as effectual* a preventive of small-pox as the old inoculation. Now, we think it has been demonstrated, beyond the possibility of contradiction, that the number of authenticated cases of small-pox after the old inoculation, and even after a former attack of the natural disease, are more numerous in proportion than those that are alleged, with any probability, of such an occurrence after complete vaccination.

"It has become a fashion among the opposers of vaccination to assert, without ceremony, and in the most positive manner, that no person ever had the small-pox twice,—and from this they conclude, without any more ado, that, if they can show one instance of its occurrence after vaccination, the question is decided in favour of the old method. We have heard the same confident assertion made in conversation; and we have therefore been at some pains to look into the evidence of the opposite proposition, which appears to us as clearly and completely established as any fact in the history of diseases. We have no longer room to insert even an abstract of those cases; but we shall refer our readers to the places where they may be found, after stating, very shortly, the two earliest that appear on record.

"The first occurred in the case of a child of Dr. Croft. 'He was inoculated by Dr. Steigerthal, physician to King George the First. Dr. Deering was an eye-witness of the operation; and assures us, great care was taken in the choice of matter. He had the small-pox of the confluent kind, and in a severe manner, in consequence of this inoculation, and yet had it again *very full,* in the natural way, twelve months after. This, says Dr. Woodville, in his History of Inoculation, p. 217, is *a striking fact, which has never been contradicted.*' A second case was published about the same time by Dr. Pierce Dod: 'It occurred in a

 son son of Mr. Richards, member of Parliament for Bridport, who was inoculated for the small-pox. About sixty pustules came out; which maturated, scabbed, and went off in the usual manner. Two years afterwards he had the disease again, more severely, in the natural way. This case was communicated to Dr. Dod by Dr. Brodrepp, a learned and experienced physician, the grandfather of the child, who attended him on both occasions,' and was much canvassed by the controversialists of that day. A third very striking case is mentioned by Mr. Ring in his Answer to Dr. Mosely (p. 209, &c.), of a person who was much seamed and scarred by natural small-pox in his youth, who, after he was a grandfather, died of a second attack of the confluent disorder. A fourth case is mentioned at p. 211, of the same work, of the very same description, and with the same issue; a fifth is detailed at p. 213; a sixth at p. 215; and a seventh at p. 280: three others are given from a foreign publication at p. 199. Several similar facts are detailed in Mr. Ring's large treatise, p. 59. 86. 946, &c.; and the case of the Earl of Westmeath's child has lately been laid before the public in a way that precludes all doubts as to its authenticity.

"On the whole, we think there are not fewer than twenty distinct cases of small-pox occurring a second time in the same subject, *each of them authenticated far more completely than any one that has been cited by the adversaries of vaccination.* We are persuaded, indeed, that we shall be supported by every impartial person who makes himself master of the whole evidence, in saying, that there are not so many as ten cases of small-pox, after perfect vaccination, proved in such a way as to be entitled to any sort of attention. Now, the Medical Council, consisting of almost all the great practitioners in London, have reported, that nearly as many persons have been already vaccinated in this kingdom, as were ever inoculated for the small-pox, since the first introduction of that practice; so that, if the two cases were exactly upon a footing, the risk of failure seems to be at least twice as great in the small-pox inoculation as in that for cow-pox.

"But the cases are not by any means on a footing; and, when rightly considered, the advantage will be found to be still more decidedly on the side of vaccination. In the first place, an infinitely greater proportion of vaccinated patients have been intentionally subjected to the most violent forms of variolous infection, than of those who had been inoculated for small-pox. For fifty years back, the confidence dence of the country, in the efficacy of inoculation, has been so firmly established, that it was seldom put to the test, either by a second inoculation, or by voluntary exposure to infection. The anxiety, and the contest about vaccination, had the effect of making it almost a regular practice to inoculate again with variolous matter, or to put the patient in some other way to the proof. It is not too much, perhaps, to say, that one fifth of the whole number vaccinated has been subjected to this severe ordeal; and that not more than one in five hundred of inoculated patients have undergone a similar probation. If the two operations, therefore, were only of equal virtue, the cases of failure should be an hundred times more numerous among the vaccinated than the inoculated patients. In point of fact, they are absolutely fewer. It deserves also to be considered, that cases of failure in inoculated small-pox must now be picked up, in a great measure, from old books or old people, and that it is fair to presume that a much greater number than can now be authenticated have occurred, and been forgotten in the course of the last seventy years; whereas, all the instances of failure in vaccination having happened within these six years, and while the keen eyes of so many disputants were fixed on the issue, it may be concluded, that few or none have been lost to the public, and that we are now completely aware of the full extent of the calamity.

"In whatever way this part of the question be considered, therefore, we conceive it to be clearly made out, that vaccination, if it do not absolutely and certainly secure the patient from the contagion of small-pox, gives him a security, at least as effectual as could be given by the old practice of inoculation. We are conscientiously persuaded, that, to this extent, it may be relied on with the most implicit confidence.

"The only other point which remains to be considered, is, whether vaccination communicates as safe and mild a disease as inoculation? Upon this, however, it would be a mere waste of words to enlarge; the public know perfectly, by experience, that the cow-pox is incomparably a milder disease than the inoculated small-pox; and there is certainly no one instance in which the fever attending it has risen to a fatal, or even to an alarming height. As to the trash that has been written to prove that it has given birth to a multitude of new and dreadful cutaneous distempers; as there is not a shadow of evidence to connect these appearances with the preceding vaccination, the only answer that can be made to it is, that it was never pretended that the cow-pox would insure the patient, for all the rest of his life, from scrophula or itch, or tinea, or leprosy, or syphilis. The whole proof that is offered, in any of the alleged cases, is, that a person who had been vaccinated, was afterwards affected by these disorders, sometimes at the distance of years.

"It was not necessary, perhaps, to make any other answer to assertions so improbable and intemperate; but Dr. Willan has condescended to answer them; and has set this part of the question, it appears to us, finally to rest. Dr. Willan, it is well known, is the oracle of the metropolis in all cutaneous disorders, and has more practice in that department than all the rest of his brethren put together. Now, he says, in the first place, that after a careful examination of all the cases alluded to, *no new disorders* have been introduced into the nosology since the discovery of vaccination; and that the old cutaneous complaints of the metropolis have not become either more virulent or more general. As a proof of this, he exhibits a table (p. 82.) of the number of cases of cutaneous eruption in the public dispensary, from 1796 to 1805; the result of which is, that their proportion to other diseases was rather greater before Dr. Jenner's discovery, than in the sixth and seventh years of vaccination. In the next place, he exhibits a statement, from the senior surgeon of the Gloucester Infirmary, in which cow-pox has been familiar for the last fifty years, which purports, 1st, That there is not a more healthy race of beings, or one more free from cutaneous complaints, than the milkers at dairies, who are constantly exposed to cow-pox; and 2d, that though many hundred patients have been under his charge for cow-pox in the last fifty years, *not one* has complained, in all that time, of any cutaneous affection as its consequence. In the last place, Dr. Willan gives it as his decided opinion, that the vaccine inoculation is much less apt to produce inflammation and suppuration of the glands than inoculated small-pox; and that he has never known an instance of scrophula that could be fairly referred to it.

"There are, no doubt, one or two unfortunate cases, and we believe no more, in which the wound in the arm has degenerated into a dangerous ulcer. This may be owing to the incautious use of a rusty lancet, or of one charged with matter which had run into putridity; or it may be owing to a singular and unaccountable irritability of constitution, akin to that which Dr. Willan says he has known produce duce the most violent disorders from the application of a blister, or give rise to incurable ulcerations from the bite of a leech. It is needless to say, that similar disasters may arise from common inoculation—they may arise from the scratch of a pin.

"Although the arguments in favour of vaccination appear, when impartially considered, to be thus evidently triumphant, we are well aware, from the recollection of our own sentiments on the occasion, that some people, who have not leisure to enter into the merits of the controversy, may be staggered by the simple and palpable fact, that a certain number of persons, of some education and acuteness, have set themselves so outrageously against it, and may think it safer to resist novelties, as to the merit of which there is a difference of opinion, and adhere to the good old way, which every body so lately concurred in recommending. To such persons, it may be of some use to state, that the good old way of inoculation of small-pox met, in its day, with an opposition not less virulent and persevering, than cow-pox seems destined to encounter; and was assailed with as much bad language, and nearly as much bad argument, as is now poured out against vaccination. Dr. Wagstaffe, in 1721, published a variety of pamphlets against it, in which he maintains, with great vehemence, that it does not prevent the small-pox in future; that it produces a variety of shocking distempers, itch, ulcers, boils, hectic, *caries*, &c.; that it often produces an unfavourable confluent small-pox; and, in general, that it is to the full as fatal as the natural disorder. The same positions were maintained in a great variety of eloquent publications by Dr. Hillary, and Messrs. Howgrave, Sparham, and Massey. But the most magnificent and imposing piece of composition that has been preserved upon this side of the question, is a sermon preached by the Rev. Edmond Massey, 'upon the dangerous and sinful practice of inoculation,' in 1722. In this performance, the reverend person maintains, that Job's distemper was the confluent small-pox, and that he had been inoculated by the Devil: he then asserts, that diseases are sent by Providence for the punishment of our sins; and that this attempt to prevent them, is, 'a diabolical operation.' He comforts himself, however, by reflecting, that its pretensions, in this way, are utterly vain and groundless;—he says they are mere 'forgers of lies,' who pretend that it will prevent the small-pox; enlarges upon the miseries and evils that inoculation threatens to introduce; and hopes that a time will come, when those preparers of poison, and spreaders of infection, will have a stigma fixed on them, and no longer be permitted to mingle with other professional men; which, he says, indeed, is as presumptuous in them as it was in the Devil to mingle with the sons of God.

"These, and similar expressions, which abound in the writings of that day, will go far, we fear, to deprive Drs. Moseley and Squirrel of any claim to originality in the style of eloquence they have exerted themselves so meritoriously to revive. We beg them, however, to believe, that it was by no means for this invidious purpose that we have referred to their prototypes, but merely with a view to set the minds of those readers at rest, who might be inclined to doubt, whether men of education could possibly be so positive and so angry in support of what was certainly wrong. Drs. Wagstaffe and Hillary, with their faithful squires and followers, have been effectually confuted by the experience of little less than a century; and their forgotten cavils and rhapsodies now excite no other emotions in the reader, than those mild sensations of contempt and wonder with which the next generation will look on the lucubrations of Squirrel and Moseley, if any accident should draw them from the shelter of that oblivion to which they are rapidly descending."

These sentiments concerning the value of vaccination appear to me just; and as they are an honourable testimonial in favour of the practice, I deemed it my duty to transcribe them at this crisis; when envy, hatred and malice are once more at work, in order to prevent parliament from recommending it to the public, and granting a further remuneration to Dr. Jenner.

The Editors of the Review pretend indeed, in their advertisement, that their publication is "*distinguished by an impartiality, which no party-zeal has hitherto called in question.*" Nothing can be more false. I do not believe that there is any publication, the impartiality of which has been so frequently or so generally called in question.

In the Anti-Jacobin Review, vol. 18, p. 417, the Edinburgh Reviewer is called *a slanderer* and *a calumniator*. He is represented, as one who *ignominiously brands the British army!* and *traduces the whole military body*. His publication is there described, as *the vehicle of false and malignant abuse, against the troops of this country*.

The same writer in the Anti-Jacobin affirms, that the object of the Edinburgh Review is, the depreciation of whatever tends to elevate, or support our country. He alludes alludes to one instance in particular, in which he represents a Critic in that Review, as taking refuge in mistatement, misquotation, and falsehood. He tells us, that in *seven* pages, the Edinburgh Reviewers tell *six deliberate falsehoods*; and asks, whether these are the critics, to whom the public are to trust, for *a faithful account of new productions*.

He asserts, that such disingenuous and fraudulent artifices must proceed from *a design to misrepresent*. He asserts, that *every person eminent for efforts beneficial to his country, may naturally expect the malignant hatred of the Edinburgh Reviewers*.

In the 19th volume of the Anti-Jacobin, we meet with the following remarks. "*Edinburgh Reviewers,—exposure of their folly and impertinence.* Mr. Godwin is unreasonably sceptical; it is, however, of no great importance; but the mention of it powerfully impels us to point out *the conscientious care*, with which, it appears, those *judges of literature, the Edinburgh Reviewers, peruse the authors whom they pretend to criticise.*

After pointing out the misrepresentations, of which the Edinburgh Reviewers are guilty, he adds, we will not insult the good sense of our readers by asking them, whether they consider this *nonsense* as *criticism*, but we would seriously advise the Edinburgh Reviewers, before they venture, in future, to give unlimited scope either to their *spleen* or their *merriment*, at least to *read* the book which they profess to analyse.

In the 20th volume of the Anti-Jacobin it is observed, that the Edinburgh Reviewers, instead of endeavouring to recommend their labours by *a faithful analysis*, and *a fair account* of the different works which they undertake to criticise, seem to have set out with the professed design of declaring war against the whole fraternity of authors. It is certain, at least, that *very few* of those whom they have honoured with their notice, have met from them with *any thing like justice*, not to mention *candour*. It is really amusing to observe the *impotent self-sufficiency, with which these critics pretend to erect themselves into judges, from whose sentence there shall be no appeal*; and it is certainly singular, that *in exact proportion*, as nearly as may be, *to the merit of an author*, is *their anxiety to vilify and degrade his work*." These are the gentlemen, whose *impartiality* has never been questioned!

The Editors of the Anti-Jacobin next remark, that in the analysis of Dr. Thompson's Chemistry, the power of the critic to injure Dr. Thompson is greatly inferior to his inclination.

inclination. He remarks, that Dr. Thompson has ascertained who this critic is; and that his conduct is the more illiberal, as he is a rival, who is endeavouring to lessen Dr. Thompson's class of pupils, in order to augment his own. It is generally understood, that the person who is suspected to be the critic, is the same who vents his spleen in the Edinburgh Medical and Surgical Journal; whose sentiments appear to be in perfect unison with those of the Editor of the Edinburgh Review, to whom he is said to be an understrapper.

Qui Bavium non odit, amet tua carmina, Mævi!

The Editors of the Anti-Jacobin observe, that in the aforesaid analysis of Thompson's Chemistry, where defects have not been *found*, they have been *made in abundance*; and that the art of *misrepresentation* has been called in to supply the deficiency of *solid grounds of censure.* They then quote two passages from Thompson's Chemistry, in order to recommend to universal notice the "*fair and conscientious impartiality*" of the Edinburgh Review. After this, it is with an ill grace the Editors of this Review tell us, their impartiality has never been called in question!

The Anti-Jacobin Reviewers inform us, that the Editor of the Edinburgh Review, who is fond of sarcasm, by leaving out half sentences, and pruning away others till they answered his purpose, "has totally altered the original meaning; and succeeded in giving the paragraph some point, *at the trifling expence of truth and candour.*" They next inform us, that the Edinburgh Reviewers are guilty of *a glaring and unprincipled falsehood*; that a friend of Dr. Thompson asked one of them, why they took such pains to detect faults, while they passed over the other parts of a work in contemptuous silence; to which he replied, "*we wish our book to sell, and know enough of the taste of the public, to suit their palate. Ridicule and invective alone are certain to command success.*"

Dr. Thompson says, he shall only take the liberty, at parting, to request of these gentlemen, not to indulge too freely in those *unjustifiable arts of criticism,* which unhappily distinguish their publication. "Any man" says he "can abuse and call names; any man can pervert and misquote the meaning of an author; but to discriminate between faults and perfections, and to point out the various degrees of merit with justice, belong only to a superior mind. If they must indulge in *severity,* let them not lose sight of *candour*. Our worthy critics ought to review this pamphlet of of mine, unless they think it better to publish a second review of the work itself. By the *usual* methods of *perversion* and *misquotation*, they may make both as ridiculous as they please."

In a subsequent part of the same volume of the Anti-Jacobin, the Editors of that work state, that the Edinburgh Review is *occasionally able*, and *uniformly abusive*; and that they have received from their Correspondents, a detection of *many of the base arts* of its writers and conductors. They propose to give an account of the Edinburgh Reviews, as they appear; but observe, that it is no pleasing task *to wade through masses of indiscriminate abuse and scurrility*; and hope, for the honour of the nation, that the second volume is already forgotten. They are of opinion, that some of the articles of that volume are, on the whole, good, and some others tolerable; but *much the greater number detestable.* After stating some of the remarks of the Edinburgh Reviewers on the Huttonian and Neptunian systems of geology, the Anti-Jacobin Reviewers observe, that a series of more unphilosophical hypotheses will not readily be found, within so small a compass, in any other work which has gained the smallest reputation, than those which are there strung together in the Edinburgh Review.

A writer in the Anti-Jacobin Review for December, 1806, says, "The Edinburgh Review is distinguished by wit and ability; and it has also these characteristic marks: First, a heresy with regard to the establishment; political and ecclesiastical, particularly the latter; and a propensity, and partiality to *every thing Scotch.* This he thinks, if not an amiable and venial defect, is nevertheless so *natural* a blemish in the work, that it proves it to be of *genuine manufacture.*"

In the next place he tells us, "it has *a caustic severity of censure*, which is not always consistent with perfect liberality and good breeding." He thinks, "WANTON PETULANCE as displeasing as the churlish moroseness of ancient pedantry." He says, he should not have noticed these imperfections of the Edinburgh Reviewers, had he not observed in the last Number, "*a more than common flow.*"——I should not have noticed them, had I not observed in their last Number a more than common degree of illiberality and injustice.

I am, &c.

JOHN RING.

